# Surface Activation of Faceted Photocatalyst: When Metal Cocatalyst Determines the Nature of the Facets

**DOI:** 10.1002/advs.201500153

**Published:** 2015-07-14

**Authors:** Bin Wang, Maochang Liu, Zhaohui Zhou, Liejin Guo

**Affiliations:** ^1^ International Research Center for Renewable EnergyState Key Laboratory of Multiphase FlowXi'an Jiaotong UniversityXi'anShaanxi710049P.R. China

**Keywords:** hydrogen production, kinetic control, metal cocatalyst, shaped photocatalyst, surface activation

## Abstract

**Pt nanoparticles with tunable size are prepared on the entire surface of facet‐engineered Cu_2_WS_4_ decahedral photocatalyst** via a kinetic‐controlled chemical reduction process. The {101} facets of the photocatalyst which featured photo‐oxidation, are successfully activated for photoreduction by Pt. The resulting photocatalyst shows an activity nine times higher compared to that of the only {001}‐facets activated catalyst obtained by a conventional in situ photodeposition route.

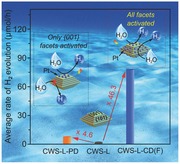

Hydrogen evolution via semiconductor‐based photocatalytic water splitting has been regarded as one of the most promising strategies to address the energy and environmental crises.^1^, ^2^, ^3^ This process has long relied on the development of a functional semiconductor photocatalyst that is both stable and active. For one of the approaches to this end, photocatalysts coupled with cocatalysts to form junction structures are attractive for use because of their unique performance on separating photogenerated charges and activating the targeted crystal surface, which, in turn, lead to the improved photocatalytic performance in terms of both stability and activity.^4^, ^5^, ^6^, ^7^, ^8^, ^9^, ^10^, ^11^, ^12^, ^13^, ^14^ Up to now, a number of materials have been employed as cocatalyst.^7^, ^8^, ^9^, ^15^, ^16^, ^17^, ^18^, ^19^, ^20^, ^21^, ^22^, ^23^, ^24^, ^25^, ^26^, ^27^, ^28^, ^29^ Yet, zero‐valent metals, such as Pt, Pd, Ru, and Ir, etc., are still the most powerful class of cocatalysts that can be both more active and selective for hydrogen production.^6^, ^9^, ^30^ The superiority can be explained by the fact of their relatively larger work function (therefore lower Fermi level) that shall more readily trap electrons.^31^

Metal cocatalysts are usually deposited by in situ photoreduction, which has a strong site‐selectivity. Such preference can be observed in a more clear way when shaped semiconductor crystals are the primary source for host materials.^32^, ^33^, ^34^, ^35^, ^36^ Generally, the resulted junction structures are considered favored because of the creditable synergy created by the specific facets of the semiconductor and the metal nanoparticles.^32^, ^33^, ^34^, ^37^ However, this method has still met with limited success to the fabrication of metal cocatalyst with controlled size and distribution, which is of crucial importance to tailoring and fine‐tuning the photocatalytic activity and selectivity. Moreover, this site‐selective process may also lead to the passivation of other unloaded surface for hydrogen evolution. Instead, a large percentage of such unloaded facets that turn to the oxidation reaction, however, may be not that necessary especially when the reaction rate is determined by reduction of H^+^.^34^, ^36^, ^38^ These issues are very important for rational design of heterojunction, thus urgent to be resolved, but challenging to date.

Herein, using well‐shaped Cu_2_WS_4_ (CWS) decahedra as model photocatalyst, and Pt as model metal cocatalyst, we systematically studied the mentioned matters. Specifically, using syringe pump and a combination of seeded growth as versatile method for kinetic control, Pt nanoparticles with tunable sizes ranging from 16.1 to 57.8 nm and adjusted distribution on the surface of CWS decahedra were successfully obtained, which in principle, enables us to regulate the surface reactive sites in terms of both number and distribution for hydrogen production. Compared with the traditional photodeposition method, the photocatalytic activity of CWS could be increased by one order of magnitude via the photo‐free chemical deposition method.

CWS decahedra enclosed by {101} facets and {001} facets were prepared according to our previously reported hydrothermal method, which involved the use of CuCl/Na_2_WO_4_/thioacetamide as precursors and water/ethanol as solvents.^34^, ^39^ The morphology could be regulated by the simply changing volume of the solvents. Related details can be found in the Experimental Section. Figure S1a,b, Supporting Information, shows the typical scanning electron microscopy (SEM) images of two kinds of CWS decahedra with altered proportions of {101}/{001}facets (designated as CWS‐S and CWS‐L, respectively). The ratios of {101}/{001} facets were roughly determined according to the process shown in Figure S2, Supporting Information. Statistic results are listed in Table S1, Supporting Information, i.e., 56%/44% and 96%/4% for CWS‐S and CWS‐L, respectively. We further investigated their light absorption ability and phase structure (Figure S3, Supporting Information). As expected, no notable difference was observed. The band gap of the CWS samples was calculated to be ≈2.1 eV according to the Kubelka–Munk method.^40^ Both CWS‐S and CWS‐L showed only tetragonal structure belonging to the *I*‐42m space group. No impurity peaks can be found. Again, the varied intensity of (101) and (002) as highlighted in Figure S3b, Supporting Information, further proves the changed {101}/{001} ratios over two samples, agreed with our SEM observations (Figure S1, Supporting Information).

Basically, photogenerated holes shall be rapidly consumed by sulfide sacrificial reagents, while the photogenerated electrons devote to reducing H^+^. According to our previous work, adjacent {101} and {001} facets were experimentally demonstrated to have different chemical potentials.^34^ The energy difference between the two adjacent {101} and {001} facets will contribute much to the charge separation, and hence enhanced oxidation and reduction during the photocatalytic process, respectively. It was found that CWS with smaller proportion of {101} facets possessed higher reactivity for hydrogen production in the presence of sodium sulfide (hole scavenger). It is therefore believed that the reduction process of the proton at the catalyst surface is the rate‐determining step. This is further confirmed by the enhanced hydrogen production over the same CWS decahedral photocatalyst containing Pt cocatalyst (accelerating reduction). In other words, for those CWS with larger {101} facets (CWS‐L), too small percentage of {001} facets to allow reduction while a majority of {101} facets is “unused” and “waste.” Exploiting of these {101} facets and activating them for H^+^ reduction is thus of high interest.

As aforementioned, the surface of a given photocatalyst can become more active by loading metal cocatalyst. Different from traditional photoreduction process which only deposit metal cocatalyst nanoparticles on {001} facets of CWS, a photo‐free chemical reduction method may allow a flexible deposition of them on the entire crystal surface without selectivity. **Figure** **1** gives a schematic of the process for a metal cocatalyst loaded by photo‐deposition and photo‐free chemical deposition, respectively, onto the surface of single‐crystal CWS decahedral photocatalyst. If light is introduced, metal ions will be only reduced on {001} facets, and photoreduction will be further accelerated due to the existence of the already deposited metal nanoparticles therein (Figure 1, right top). On the contrary, when the reaction occurs without photoirradiation, metal ions reduced by chemical reductant will nucleate at either {101} or {001} facets. In this case, photogenerated electrons can also transfer to {101} facets, then to metal nanoparticles and react with H^+^ (Figure 1, right bottom). We therefore expect a further improvement of hydrogen evolution rate by involving the {101} facets for possible hydrogen production. More importantly, by alternatively changing the injection rate of metal ion precursor or the reduction capacity of the solution, that is, regulating the reaction kinetics, the particle size of these metal particles on the surface of CWS decahedron can be precisely tuned. This notion renders us to investigate the correlation of the size effects of metal cocatalyst with their associated catalytic properties.

**Figure 1:**
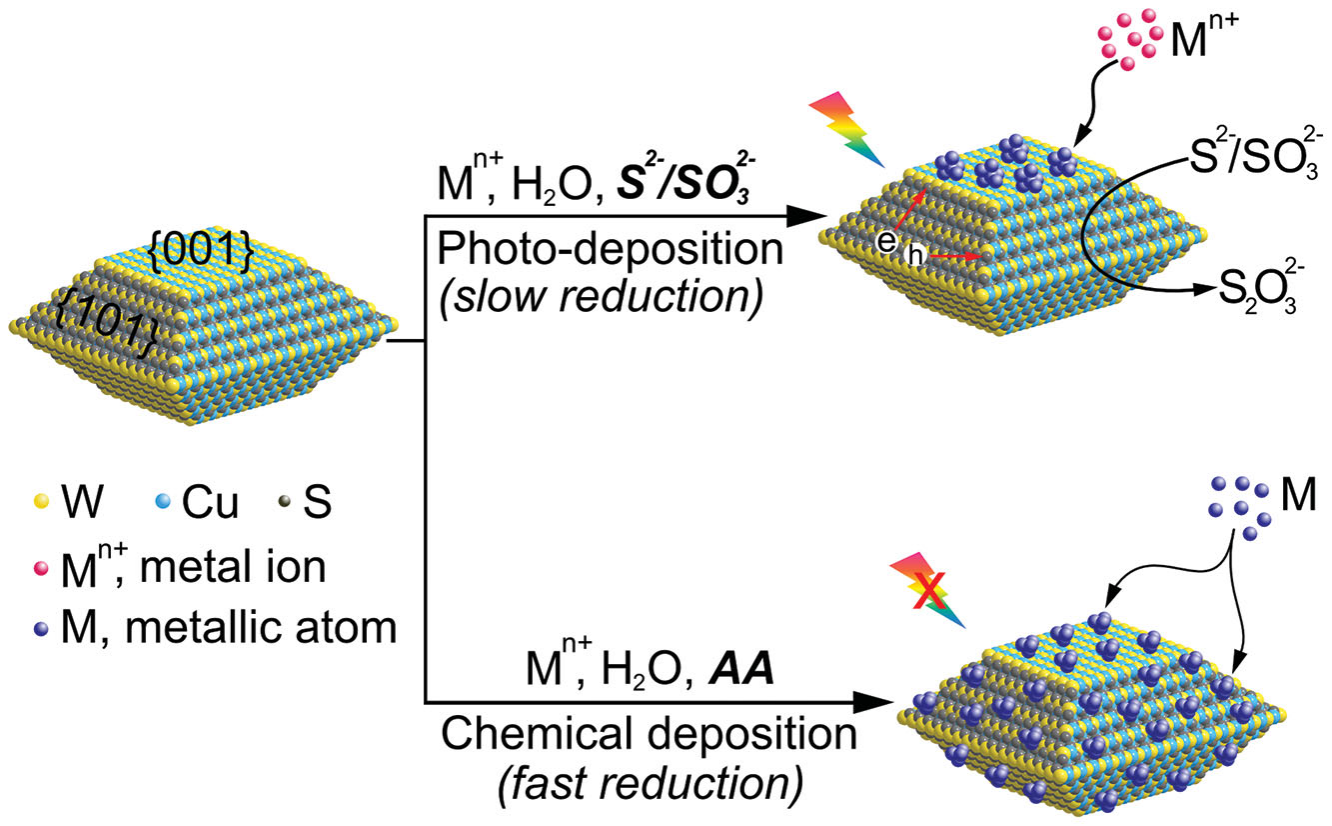
Schematic illustrations of metal cocatalysts deposited on a CWS single crystal by photodeposition and chemical deposition methods. Because of the separation of photogenerated electrons and holes, metal nanoparticles will only accumulate at {001} facets during photoassisted slow reduction (right top). While during the photo‐free, AA‐assisted fast reduction process, these metal nanoparticles will disperse at both {001} and {101} facets (right bottom) without selectivity. The resulting products will intrinsically show different photocatalytic manners for hydrogen evolution.

Our syntheses were carried out in an aqueous solution with CWS‐S as photocatalyst, Na_2_S and Na_2_SO_3_ as sacrificial reagent, in the presence of visible light irradiation or using ascorbic acid (AA) as reductant. Generally, Na_2_S and Na_2_SO_3_ have been widely used in such reaction because they would act as hole scavengers (S^2−^ + SO_3_^2−^ + 2h^+^ = S_2_O_3_^2−^) to avoid possible photocorrosion of chalcogenide photocatalysts.^9^, ^22^, ^30^, ^41^, ^42^, ^43^, ^44^ More importantly, such sulfur compounds can be largely obtained from petrochemical industries or nature resources.^2^ Metal precursor was then introduced by one‐shot injection or slow addition using a syringe pump. **Figure** **2** shows the representative SEM images of the corresponding Pt loaded CWS photocatalysts. As shown in Figure 2a, Pt nanoparticles with an average size of ≈ 47.3 nm (Figure S4a, Supporting Information, for statistics of size distribution) mainly occurred on the {001} facets through photo‐deposition. On the other hand, Pt nanoparticles randomly covered both {101} and {001} facets of the CWS decahedra when they were reduced by AA without light irradiation (Figure 2b–d). Particularly, the mean size of Pt particles could be controlled from ≈16.1 to ≈57.8 nm by controlling the injection rate of ammonium chloroplatinate ((NH_4_)_2_PtCl_6_) (Figure S4b–d, Supporting Information). In principle, during this kinetic controlled chemical reduction, (NH_4_)_2_PtCl_6_ molecules will be quickly reduced in to their metallic species once they encounter with the aqueous solution as the amount of AA is much higher than the stoichiometric ratio. The nucleation and growth of Pt nanoparticles therefore is determined by the concentration of Pt monomers formed. For example, when (NH_4_)_2_PtCl_6_ is one‐shot injected, the initial concentration of Pt monomers is very high. While most of these monomers are nucleated on the surface of CWS decahedra, few of them undergo further growth in solution. As a result, Pt nanoparticles with very small sizes can be obtained (Figure 2b). If (NH_4_)_2_PtCl_6_ is introduced slowly by a syringe pump, there are only small amount of monomers produced at the beginning, therefore the number of nucleus is significantly decreased. Lately formed Pt monomers, instead of nucleation, will grow on to these initial nuclei, resulting very large Pt nanoparticles (Figure 2d). Similar arguments can be applied when the injection rate is between the two extremes (Figure 2c), as well as in the photodeposition process (Figure 2a). In order to further confirm that the Pt nanoparticles could be deposited on the entire facets of the decahedral CWS, STEM‐EDXS elemental mapping analysis were taken on sample CWS‐S(F) (see **Figure** **3**). Typical transmission electron microscopy (TEM), scanning transmission electron microscopy (STEM), and the combined images (Figure 3a,b), clearly show different contrast between the host photocatalyst and cocatalyst. Further investigation at the magnified area in Figure 3c, by an energy‐dispersive X‐ray spectrometer (EDXS) analysis (Figure 3d–i) provides a direct evidence of the uniformly composed CWS, and well‐dispersed Pt nanoparticles on both {101} and {001} facets. Cu was also found outside the host material (Figure 3g). This phenomenon may be a result of the unstable manner of CWS under the exposure of strong TEM electron beam. In addition, X‐ray photoelectron spectroscopy (XPS) analysis also was employed to determine the chemical states of these Pt nanoparticles. As shown in Figure S5, Supporting Information, both samples were found with Pt(0) as the major product. A small amount of Pt(II) were also detected in the two samples. The trace amount of Pt(II) is considered to be a result of the formation of Pt–S bond at the surface of the photocatalyst, which is necessary for the intermediate connection of CWS and Pt nanoparticles. It is also worth pointing out that the XPS peak ratios are a little different for the two samples. This deviation may be a result of the altered number of Pt–S bond which is dependent on the size of Pt nanoparticles thus the interfacial area of the two materials.

**Figure 2:**
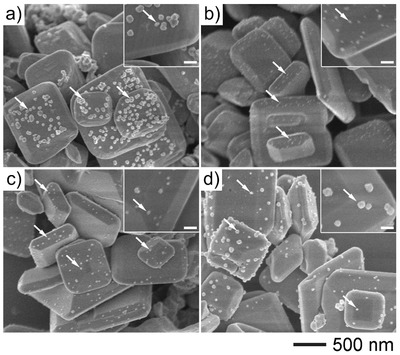
SEM images of 1 wt% Pt loaded CWS‐S photocatalysts. Pt nanoparticles were deposited by a) photoreduction, and b–d) chemical reduction of (NH_4_)_2_PtCl_6_ with (b) one‐shot fast injection, (c) moderate injection, and (d) slow injection of an (NH_4_)_2_PtCl_6_ aqueous solution. The scale bar in the inset (a) is 100 nm and also applied to the other insets. White arrows indicate the representative Pt nanoparticle.

**Figure 3:**
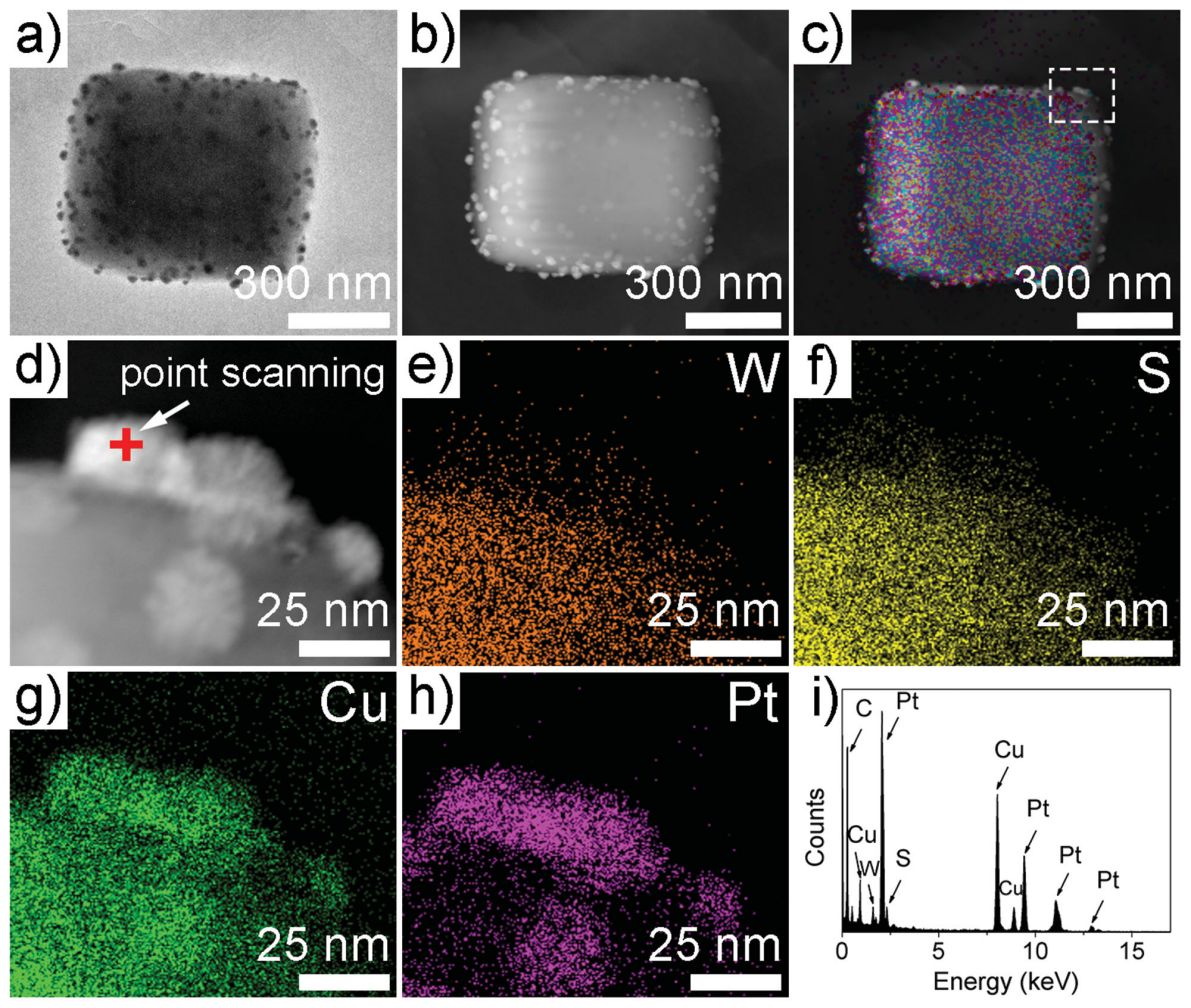
a) TEM images of a CWS‐S single decahedral crystal that is coupled with chemically deposited Pt nanoparticles (CWS‐S‐CD(F)), b) the corresponding STEM images, c) the phase map elemental distribution on the CWS‐S single crystal, d) the magnified STEM images of the selected area shown in (c), e–h) W, S, Cu, Pt elemental map of (d), respectively. i) EDXS spectrum of the point labeled in (d). The existence of Pt nanoparticles on both {001} and {101} facets were clearly validated.

We also checked the XRD property of these Pt loaded CWS‐S samples (Figure S6, Supporting Information). In addition to the unchanged crystallinity of the host material, peaks belonging to Pt cannot be found which should be attributed to the small amount of loaded Pt (1 wt%) and their small size. Taken together, we have successfully demonstrated the feasibility of chemical reduction for surface active sites fabrication.

Theoretically, the electronic energy structures heavily rely on the surface atomic configuration. According to our previous report using first‐principles calculation, {001} facets and {101} facets showed a type‐II band alignment, with the band offsets of 80 and 60 meV for conduction band and valence band, respectively.^34^ **Figure** **4** provides the corresponding band structures and the plausible photocatalytic process over different Pt loaded CWS photocatalysts. In brief, after photoexcitation, the generated electrons move to Pt nanoparticles to conduct reduction of H^+^, while the holes transfer to uncovered {101} facets to oxidize Na_2_S/Na_2_SO_3_ sacrificial reagent. Compared with Pt on {001} facets, higher potential is available for excited electrons jumping from {101} facets to Pt nanoparticles, enabling the faster interfacial charge transfer. Consequently, Pt nanoparticles on {101} facets is believed to possess better capacity in comparison to their {001}‐facets counterpart for hydrogen production.

**Figure 4:**
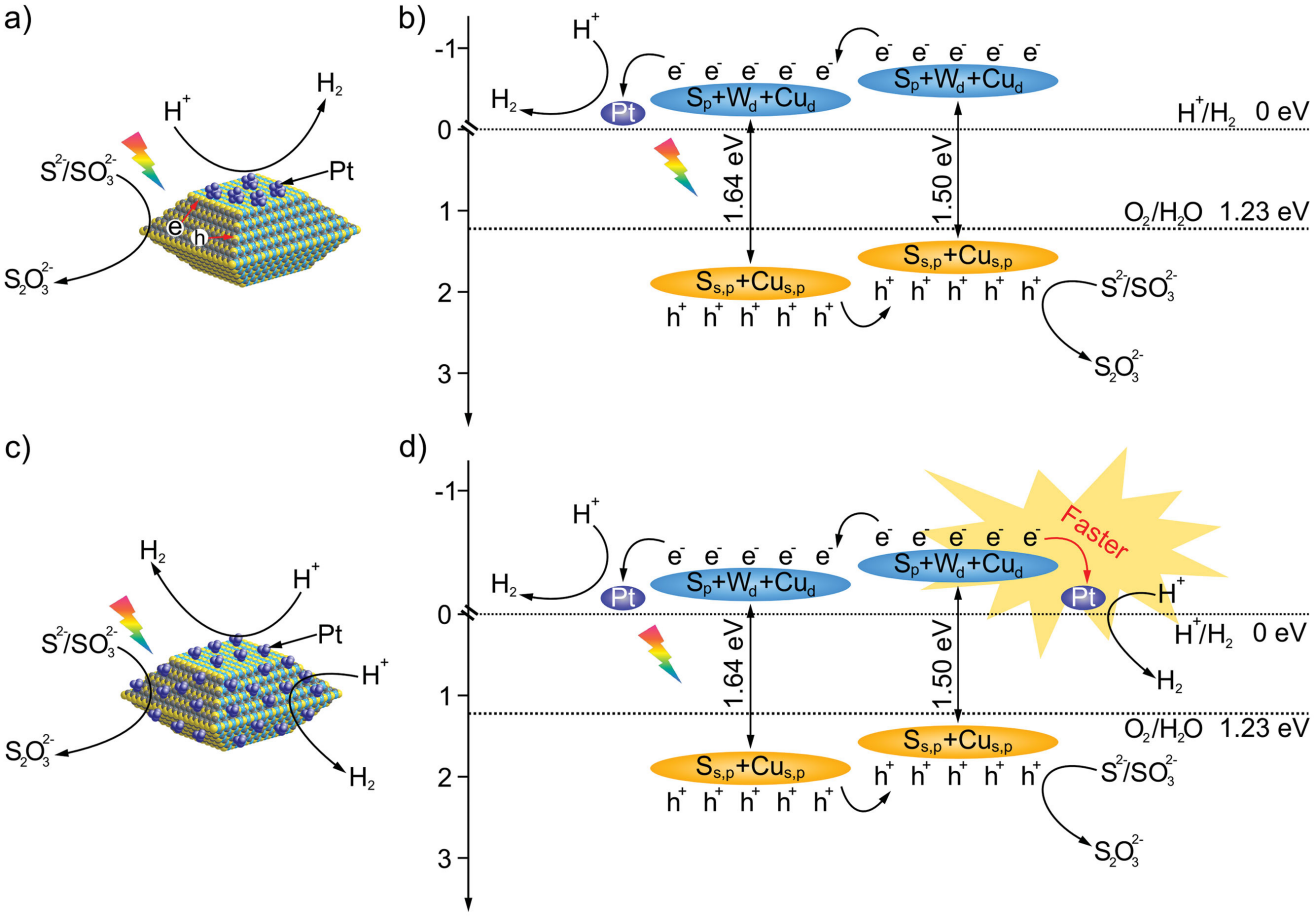
Photocatalytic mechanism of Pt loaded CWS photocatalyst with Pt deposited via a,b) photoreduction, and c, d) chemical reduction processes. Clearly, {101} facets, which only serve as the oxidation sites during the photoreduction process, will be activated for reduction during the chemical reduction process, leading to the enhanced activity for H_2_ evolution.

We then carried out a series of photocatalytic experiments to verify above rationales. Typically, the reaction was placed in an aqueous solution containing aforementioned photocatalysts, Na_2_S, and Na_2_SO_3_. Visible light was introduced from a 300 W Xe lamp coupled with a 420 nm cut‐off filter. The amount of H_2_ was measured using a gas chromatography. **Figure** **5**a shows the average rate of hydrogen evolution over the various Pt loaded CWS‐S photocatalysts. Obviously, CWS‐S with both {101} facets and {001} facets covered by Pt nanoparticles presents higher photocatalytic activity than only {001}‐sensitized one (with same amount of Pt, i.e., 1 wt% loaded). This enhancement can reasonably attribute to the activation of {101} facets for hydrogen reduction. Despite of the little decreased photoreactivity with the gradually increased size of these chemically deposited Pt nanoparticles, the superiority of this new synergy is clearly demonstrated. We further examined the photocatalytic hydrogen performance over the CWS decahedra with different proportion of {101}/{001}, i.e., CWS‐S, CWS‐L (Figure 5b). For pure CWS or CWS with photodeposited Pt (Figure S7a b, Supporting Information, SEM images of CWS‐S and CWS‐L, respectively), since charge separation is dominated by adjacent {001} and {101} facets, H_2_ molecules are expected to evolve preferentially from {001} facets. This preference gave the higher photocatalytic activity obtained over the host photocatalyst with enlarged {001} facets, i.e., CWS‐S photocatalyst. On the contrary and much more interestingly, when both {001} and {101} facets were activated by chemical deposited Pt nanoparticles, CWS‐L decahedra (Figure S7d, Supporting Information) exhibited more than twice of the activity for hydrogen production compared with CWS‐S decahedra (Figure S7c, Supporting Information), with hydrogen evolution rates of 161.6 versus 76.8 μmol h^−1^. The results were futher validated by time‐coursed hydrogen production of specific activities. Specifically, BET specific surface areas of CWS‐S and CWS‐L photocatalysts were determined to be 1.767 and 1.982 m^2^ g^−1^, respectively (Table S1, Supporting Information). The photocatalytic properties over the 1 wt% Pt loaded CWS‐S and CWS‐L photocatalysts, normalized with respect to their specific surface areas, were thus obtained and demonstrated the same activity order (Figure 5c). As aforementioned, Pt sensitized {101} facets will become practically feasible for hydrogen reduction. More importantly, the elevated conduction band of {101} facets (compared with {001} facets) permits a higher transferring efficiency of electrons from CWS to Pt. This synergic effect indicates that larger {101} facets, namely CWS‐L photocatalyst should be more favored for improving the activity of hydrogen production. Considering the close size distribution of the Pt nanoparticles loaded by same method, i.e., around 50 nm by photoreduction, and around 16 nm by chemical reduction (see Figure S8, Supporting Information, for statistic results of size distribution of Pt nanoparticles), our photocatalytic hydrogen production over the two kinds of CWS decahedral photocatalysts clearly provided the direct proof on the superior ability for H^+^ reduction by the Pt activated {101} facets. In addition, We also evaluated the stability of the 1 wt% Pt loaded CWS‐L‐CD(F) photocatalyst. As shown in Figure 5d, the photocatalyst is generally stable. The slight decrease in activity after a time might be a result of the depletion of the sacrificial reagents and the generation of byproducts such as S_2_O_3_^2−^. In short, the above results thus provide a model demonstration of surface activation for hydrogen evolution by taking advantage of well resolved shaped semiconductors coupled with kinetically controlled metal cocatalyst nanoparticles.

**Figure 5:**
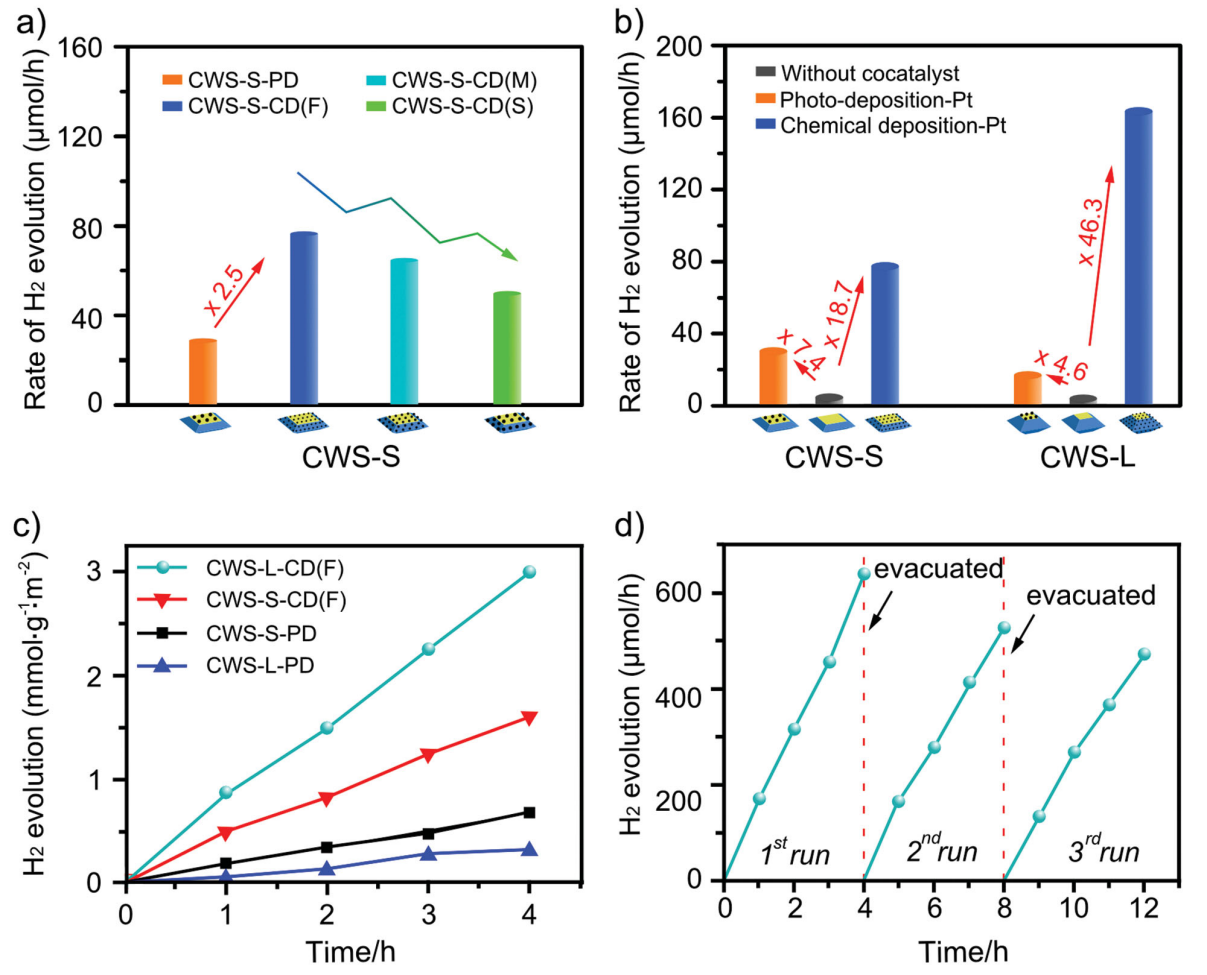
a) Rates of photocatalytic hydrogen production over the various 1 wt% Pt loaded CWS‐S photocatalysts. These CWS‐S photocatalysts coupled with chemically deposited Pt nanoparticles clearly shows the advantageous photocatalytic activity over that coupled with photodeposited Pt nanoparticles. b) Comparative study over the facet‐ratio effect on the photocatalytic properties of the CWS photocatalysts. Interestingly, using the same methods for loading Pt nanoparticles, different photocatalytic behaviors of the two sets of photocatalysts were observed, indicating the altered synergic effects induced by CWS crystal facets and Pt nanoparticles. c) Time‐coursed photocatalytic activities that were normalized to specific surface areas over the two sets of CWS photocatalysts. d) Time‐coursed photocatalytic hydrogen production over CWS‐L‐CD(F) decahedra. Reaction conditions: 0.1 g catalyst, 220 mL aqueous solution containing 0.35 mol L^−1^ Na_2_S and 0.25 mol L^−1^ Na_2_SO_3_, side irradiation Pyrex cell, cutoff filter (λ ≥ 420 nm), 300 W Xe lamp. All the tests were repeated at least three times to ensure the reproducibility, which also gave the mean rates of H_2_ evolution.

In summary, Pt nanoparticles were successfully loaded on both {001} and {101} facets of CWS decahedral photocatalysts through a facile chemical reduction methods. It was found that large percentage of {101} facets were activated for hydrogen evolution by this method, giving rise to the significantly improved hydrogen production activity. In additional to the size effect of the Pt nanoparticles, photoexcited electrons were found to be more active on Pt sensitized {101} facets, arising from their larger band level difference. The activity of the all‐surface activated CWS‐L photocatalyst was found to be one order of magnitude higher than that with only {001}‐facets activated. This work therefore provides an alternative strategy for the rational fabrication of surface active sites, with controlled number and sizes. The method is also expected to be readily extended to other kinds of photocatalysts.

## Experimental Section

*Chemicals and Materials*: Sodium tungstate dihydrate (Na_2_WO_4_·2H_2_O), cuprous chloride (CuCl), thioacetamide (TAA), sodium sulfide (Na_2_S), sodium sulfite (Na_2_SO_3_), ethanol (CH_3_CH_2_OH), and ascorbic acid (AA) were used are purchased from Sinopharm Chemical Reagent Co., Ltd. Ammonium hexachloroplatinate [(NH_4_)_2_PtCl_6_] is offered by Alfa Aesar, A Johnson Matthey Company. All the materials were analytical grade and used without further purification. The water used in all syntheses was deionized water with a resistivity of 18.25 MΩ cm.

*Preparation of Cu_2_WS_4_ (CWS) Decahedra*: CWS was prepared by hydrothermal synthesis according to our previous report.^34^, ^39^ Typically, Na_2_WO_4_·2H_2_O (0.005 mol), CuCl (0.01 mol), and thioacetamide (0.025 mol) were added to the solution containing deionized water (30 mL) and ethanol (30 mL) with magnetic stirring to form a homogeneous suspension. The reaction mixture was then sealed in a 100 mL capacity Teflon‐lined stainless steel autoclave, followed by heat treatment at 200 °C for 72 h. After cooling, the resulted precipitates were centrifuged and washed with ethanol and deionized water for several times, respectively, and dried under vacuum at 60 °C for 8 h. CWS decahedra prepared in this standard process were found to have a small proportion of {101} facets (see Figure S1a, Supporting Information, designated as CWS‐S); If we use deionized water (20 mL) and ethanol (20 mL) in the standard synthesis, the obtained CWS decahedra will have a large percentage of {101} facets (see Figure S1b, Supporting Information, designated as CWS‐L).

*Preparation of Pt Coupled CWS Photocatalyst with Pt Loaded by In Situ Photodeposition*: Pt (1 wt%) was selected as a cocatalyst for the promotion of hydrogen evolution. Typically, the as‐prepared CWS was dispersed by a magnetic stirrer in an aqueous solution (220 mL) containing Na_2_S (0.35 m) and Na_2_SO_3_ (0.25 m), a calculated amount of (NH_4_)_2_PtCl_6_ was then added into solution. The obtained suspension was irradiated by Xe lamp for 1 h. After reaction, the suspension was filtered, washed with deionized water for several times, and dried at 60 °C for 8 h in a vacuum oven. The obtained sample was designated as CWS‐S‐PD or CWS‐L‐PD, depending on the facet‐ratio of CWS.

*Preparation of Pt Coupled CWS Photocatalyst with Pt Loaded by Kinetic‐Controlled Chemical Deposition*: The synthesis is similar to the in situ photoreduction process expect that the reduction was precisely controlled by a syringe pump and without light irradiation. In detail, the as‐prepared CWS was dispersed by a magnetic stirrer in an aqueous solution (150 mL) containing a certain amount of ascorbic acid as reductant and without light irradiation. Then, 50 mL solution containing a calculated amount of (NH_4_)_2_PtCl_6_ was introduced in the reaction by one‐shot injection (fast) or at a speed of 300 mL h^−1^ (moderate) or 100 mL h^−1^ (slow). The suspension was continuously magnetically stirred for 5 h. Finally, the obtained product was centrifuged, washed with deionized water for several times, and dried at 60 °C for 8 h in a vacuum oven. The molecules ratio of ascorbic acid to (NH_4_)_2_PtCl_6_ was 100:1. The obtained samples were designated as CWS‐S‐CD(F), CWS‐S‐CD(M), and CWS‐S‐CD(S), or CWS‐L‐CD(F), CWS‐L‐CD(M), and CWS‐L‐CD(S), respectively. F, M, and S represent “fast,” “moderate,” and “slow,” respectively.

*Photocatalytic Reaction*: Photocatalytic reactions of hydrogen production from water were conducted in a gas‐closed system with a side irradiation Pyrex cell. 100 mg of photocatalyst powder was dispersed into solution (220 mL) containing 0.35 mol L^−1^ Na_2_S and 0.25 mol L^−1^ Na_2_SO_3_ by a magnetic stirrer. After being evacuated by N_2_ gas. The suspension was irradiated by visible light (λ ≥ 420 nm) through a cutoff filter from a 300 W Xe lamp. The amount of H_2_ gas was determined using a gas chromatography of Bruker GC‐450. Each test was conducted at least three times to confirm the reproducibility, which also gave the mean activity of the photocatalyst.

*Characterization*: The X‐ray diffraction (XRD) patterns were obtained from a PANalytical X'pert MPD Pro diffractometer operated at 40 kV and 40 mA using Ni‐filtered Cu Kα irradiation (Wavelength 1.5406 Å). UV–vis absorption spectra were measured on a HITACHI U4100 instrument equipped with labsphere diffuse reflectance accessory using BaSO_4_ as the reference. A JSM‐7800F instrument (JEOL) was used to perform field‐emission scanning electron microscopy (FE‐SEM). The TEMimages and STEM images were obtained from a FEI Tecnai G2 F30 S‐Twin transmission electron microscope at an accelerating voltage of 300 kV. An OXFORDMAX‐80 energy‐dispersive X‐ray spectrometer which was mounted in the above TEM was used to conduct elemental analysis. N_2_ adsorption–desorption isotherms were conducted at 77 K using an Accelerated Surface Area and Porosimetry Analyzer (ASAP 2020, Micromeritics) after degassing the samples at 150 °C for 2 h. Surface area was determined using the Brunauer–Emmett–Teller (BET) methods. XPS measurements were conducted on a Kratos spectrometer (AXIS Ultra DLD) with monochromatic Al Kα radiation (*hν* = 1486.69 eV), and with the pressure of sample analysis chamber under high vacuum (<3 × 10^−9^ Torr). All binding energies were referenced to the C 1s peak at 284.8 eV.

## Supporting information

As a service to our authors and readers, this journal provides supporting information supplied by the authors. Such materials are peer reviewed and may be re‐organized for online delivery, but are not copy‐edited or typeset. Technical support issues arising from supporting information (other than missing files) should be addressed to the authors.

Supplementary
