# Supplementary material for: Surface Activation of Faceted Photocatalyst: When Metal Cocatalyst Determines the Nature of the Facets
Source: Adv Sci (Weinh). 2015 Jul 14;2(11):1500153. doi: 10.1002/advs.201500153 (PMC5115336; doi:10.1002/advs.201500153)
Supplement: Supplementary file 1 — Supplementary [file ADVS-2-0d-s001.pdf]

Copyright WILEY-VCH Verlag GmbH & Co. KGaA, 69469 Weinheim, Germany, 2015.

ADVANCED SCIENCE
Open Access

Supporting Information

for *Adv. Sci.*, DOI: 10.1002/advs. 201500153

Surface Activation of Faceted Photocatalyst: When Metal Cocatalyst Determines the Nature of the Facets

Bin Wang, Maochang Liu, Zhaohui Zhou, and Liejin Guo**

Supporting Information

Surface Activation of Faceted Photocatalyst: When Metal Cocatalyst Determines the Nature of the Facets

Bin Wang, Maochang Liu, *Zhaohui Zhou, LiejinGuo**

Table S1. Average percentages of {101} (P_{101}) and {001} (P_{001}) facets, calculated from the surface area of exposed facets from SEM images, and BET surface areas of the two CWS samples.

Sample	P_{101}	P_{001}	$R = P_{101}/ P_{001}$	BET surface area(m^2/g)
CWS-S	56%	44%	56/44	1.767
CWS-L	96%	4%	96/4	1.982

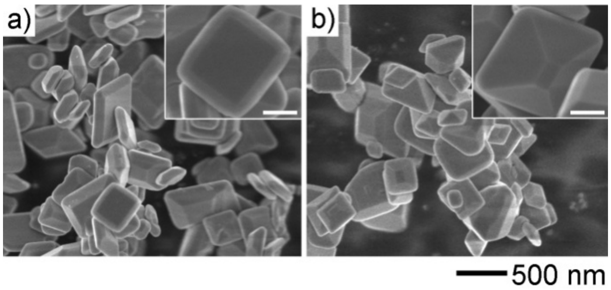

Figure S1. The SEM images of (a) CWS-S and (b) CWS-L. Insets are corresponding magnified SEM images of a single particle. Scale bars in the insets are 200 nm.

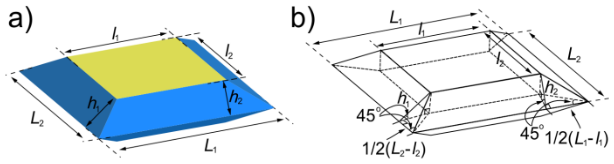

Calculation for the ratio (R) of {101} to {001}:

$h_1 = 1/2 \times (L_2 - l_1) \times \tan 45°$;

$h_2 = 1/2 \times (L_1 - l_1) \times \tan 45°$;

$R = (4 \times 1/2 \times (L_2 + l_2) \times h_1 + 4 \times 1/2 \times (L_1 + l_1) \times h_2) / (2 \times l_1 \times l_2)$

Figure S2. (a) Three-dimensional models and (b) corresponding wire-frame model of a CWS decahedra.

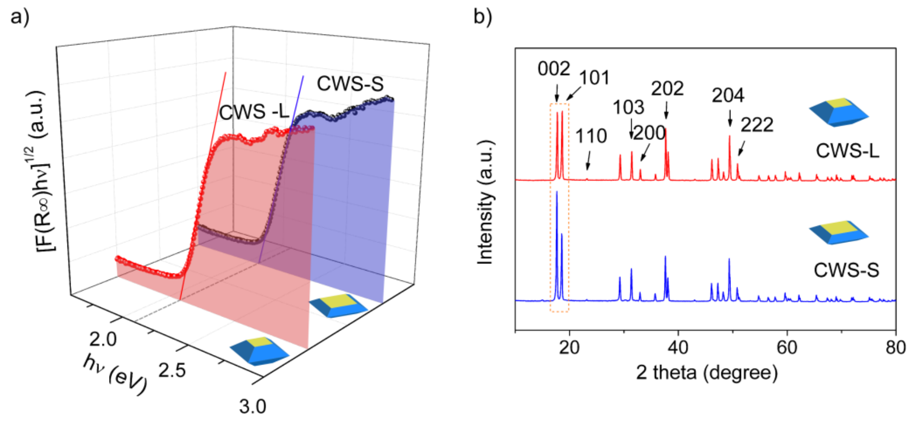

Figure S3. (a) K-M curves obtained by the UV-Vis absorption spectra and (b) XRD patterns of sample CWS-S and (b) CWS-L.

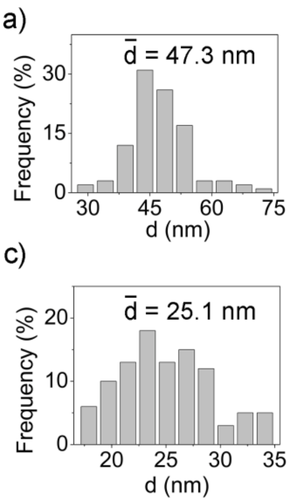

Figure S4. Size distributions of Pt nanoparticles on different Pt coupled CWS-S photocatalysts. (a) CWS-S-PD, (b) CWS-S-CD(F), (c) CWS-S-CD(M), and (d) CWS-S-CD(S), respectively.

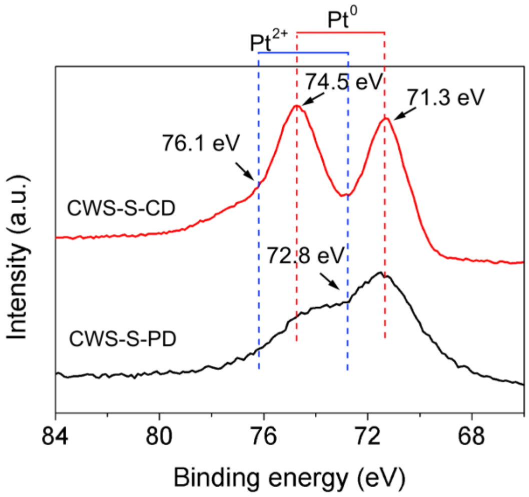

Figure S5. XPS spectra of Pt 4f in CWS-S-PD and CWS-S-CD. Clearly, most of them are in the form of Pt(0), while a trace amount of Pt(II) was also detected.

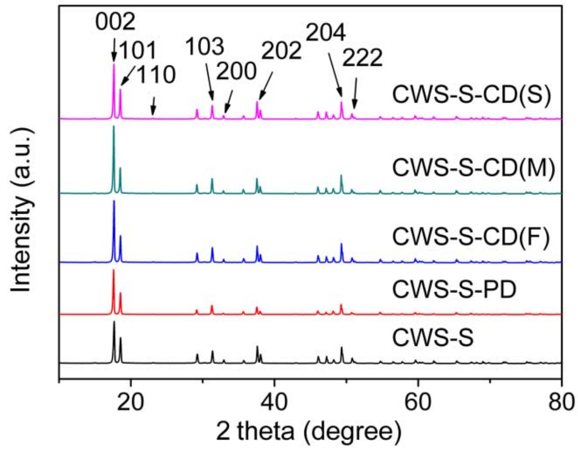

Figure S6. XRD patterns of pure CWS-S photocatalyst and that after Pt nanoparticles were loaded by photo-deposition or chemical deposition methods.

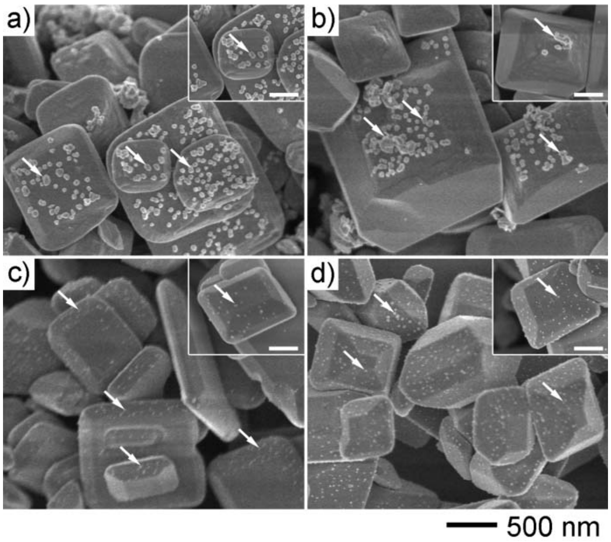

Figure S7. SEM images of (a, c) CWS-S and (b, d) CWS-L photocatalysts after Pt nanoparticles were (a, b) photo-deposited and (c, d) chemically deposited, respectively. Scale bars in the insets are 300 nm. White arrows indicate the representative Pt nanoparticle.

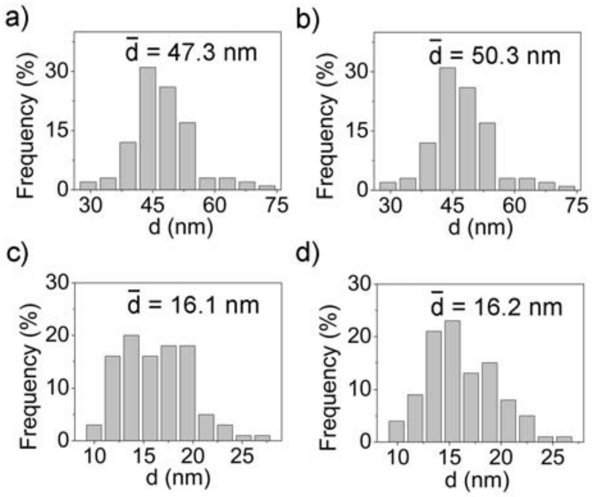

Figure S8. Size distributions of Pt nanoparticles on different Pt coupled CWS photocatalysts. (a) CWS-S-PD, (b) CWS-L-PD, (c) CWS-S-CD(F), and(d) CWS-L-CD(F), respectively.
